# Philip H. Austen

**Published:** 1878-11

**Authors:** 


### OBITUARY.

*Philip H. Austen, A. M., M. D., D. D. S.*, entered into his rest on the morning of October 28th, 1878, at his residence, No. 8 Cathedral Street, Baltimore City, in consequence of a burn received on the 1st inst. Professor Austen had for a long time previous to the accident which terminated so fatally, been in ill health, but was recovering somewhat from the most serious symptoms of his affection which was nervous in its character. Early in the morning of October 1st, Prof. Austen was aroused by a noise which caused him to suppose that burglars were in the house, and on going down stairs to inspect the lower rooms he made a mis-step, and the candle which he carried was brought in contact with his night dress. His lower limbs were immediately enveloped in flames, but with great presence of mind he hurriedly returned to his study and obtained a shawl which he remembered was there, and wrapping himself in it succeeded in extinguishing the flames without alarming his family. He was, however, seriously burned, and owing to his weak condition, the exhaustion resulting from his injuries was too great to be overcome by the great vitality and elasticity of spirits for which he was noted, and death occurred after some weeks of suffering.

Prof. Austen was a remarkable man, and one of the best informed on all general subjects. His talents were of the highest order, and his indomitable will and perseverance, together with his great intelligence and well stored mind, ensured success in all his literary undertakings. He was born in Baltimore City, in 1822, and graduated at Yale College when but 19 years of age. Two years later he graduated in Medicine at the University of Maryland School of Medicine, and practiced medicine in Baltimore City for about ten years. He then turned his attention to Dentistry, and graduated in the Baltimore College of Dental Surgery in 1849. In the same year he was appointed Lecturer on Chemistry and Metallurgy in his Alma Mater, and in 1852 was elected Professor of Dental Mechanism. This position he very acceptably filled until 1860, when he was elected Professor of the Principles of Dental Science and Chemistry, and occupied this chair until 1875, when ill health and a determination to remove from the City and engage in active out-door business, compelled him to sever his long connection with the Baltimore College of Dental Surgery. He was also the Dean of this College from 1853 to 1865. After leaving the Baltimore College, he occupied the position of Civil Engineer in directing the working of the Preston County, West Virginia Coal and Iron Mines, of which he was part owner, and which, in consequence of his great ability, were called "The Austen Mines."

Prof. Austen while in West Virginia, was untiring in his charitable efforts to improve the condition of the people of the neighborhood in which he resided, and during a scourge of typhoid fever endeared himself to the sufferers by his constant attention and gratuitous medical services. As an author, Prof. Austen had considerable reputation, being the author of the translation of Jourdain's work on "Diseases and Surgical Operations of the Mouth." He also edited, in connection with Profs. Gorgas and Latimer, the last edition of "Harris' Principles and Practice of Dentistry," which is used as a text book in all of the reputable Dental Colleges. Prof. Austen was a consistent member of the Presbyterian Church, and leaves a family consisting of a wife and three daughters. To the education of his daughters he devoted a great deal of time and with the greatest success, as he was an accomplished scholar, and displayed a rare tast for literature and the ancient and modern languages.

While in dental practice, his attention was chiefly given to dental mechanism, in which he displayed the greatest skill, being second to no other practitioner, and his lectures on the same subject were replete with useful information and scientific details. He was a fluent speaker, and was always an interesting and instructive lecturer. He was also a prominent member of the Maryland Academy of Sciences.

